# Advancing the automation of plant nucleic acid extraction for rapid diagnosis of plant diseases in space

**DOI:** 10.3389/fpls.2023.1194753

**Published:** 2023-06-14

**Authors:** Natasha J. Haveman, Andrew C. Schuerger, Pei-Ling Yu, Mark Brown, Robert Doebler, Anna-Lisa Paul, Robert J. Ferl

**Affiliations:** ^1^ NASA Utilization & Life Sciences Office (UB-A), Kennedy Space Center, Merritt Island, FL, United States; ^2^ Department of Plant Pathology, University of Florida, Space Life Science Lab, Merritt Island, FL, United States; ^3^ Department of Plant Pathology, University of Florida, Gainesville, FL, United States; ^4^ Claremont BioSolutions Limited Liability Company (LLC), Upland, CA, United States; ^5^ Department of Horticultural Sciences, University of Florida, Gainesville, FL, United States; ^6^ Interdisciplinary Center for Biotechnology Research, University of Florida, Gainesville, FL, United States; ^7^ University of Florida Office of Research, University of Florida, Gainesville, FL, United States

**Keywords:** automation, spaceflight, plant nucleic acid extraction, plant disease diagnosis, nanopore sequencing

## Abstract

Human space exploration missions will continue the development of sustainable plant cultivation in what are obviously novel habitat settings. Effective pathology mitigation strategies are needed to cope with plant disease outbreaks in any space-based plant growth system. However, few technologies currently exist for space-based diagnosis of plant pathogens. Therefore, we developed a method of extracting plant nucleic acid that will facilitate the rapid diagnosis of plant diseases for future spaceflight applications. The microHomogenizer^™^ from Claremont BioSolutions, originally designed for bacterial and animal tissue samples, was evaluated for plant–microbial nucleic acid extractions. The microHomogenizer^™^ is an appealing device in that it provides automation and containment capabilities that would be required in spaceflight applications. Three different plant pathosystems were used to assess the versatility of the extraction process. Tomato, lettuce, and pepper plants were respectively inoculated with a fungal plant pathogen, an oomycete pathogen, and a plant viral pathogen. The microHomogenizer^™^, along with the developed protocols, proved to be an effective mechanism for producing DNA from all three pathosystems, in that PCR and sequencing of the resulting samples demonstrated clear DNA-based diagnoses. Thus, this investigation advances the efforts to automate nucleic acid extraction for future plant disease diagnosis in space.

## 1 Introduction

The goal of establishing a sustained human presence, first in space and then on the Moon and Mars, involves humanity’s ability to create a habitable environment with resources to support life. Plants serve as vital resources that could check many of the boxes needed to sustain manned missions in space. They could provide food, oxygen, and water through regenerative life support systems (BLSS) or as a resource for other raw products and medicine (Wheeler, 2011; McNulty et al., 2021; De Micco et al., 2022; Haveman et al., 2022; Verseux et al., 2022). As humanity develops various infrastructures, technologies, and knowledge to ensure successful plant cultivation beyond Earth, the availability of simplified methods to determine plant health and wellness in extraterrestrial environments is a challenge that needs to be addressed. The spaceflight environment presents plants with unique stresses that can impact their development, adaptation, and interaction with microorganisms, all of which could affect their ability to thrive (Ryba-White et al., 2001; Paul and Ferl, 2002; Foster et al., 2014; Schüler et al., 2015; Johnson et al., 2017; Sng et al., 2019; Califar et al., 2020; Medina et al., 2021; Schuerger, 2021; Schuerger et al., 2021; Hughes and Kiss, 2022; Schuerger et al., 2022). Thus, a point-of-care (POC) protocol to monitor plant health and rapidly diagnose plant diseases is critical for identifying management modalities for the safe production and consumption of agricultural products.

Advances in technology, driven largely by portability, have increased the demand for onsite plant disease diagnosis among field specialists, crop consultants, and growers (Ali, 2022). Rapid disease identification is critical to preventing further spread and substantial losses in the multibillion-dollar agricultural industry each year (Oerke, 2006; Jaganathan et al., 2018). The current methods for onsite plant disease diagnosis center around direct and indirect methods of detecting the properties of pathogens. Indirect methods analyze the impacts of the pathogens on the physiological plant responses; these include techniques such as spectroscopic and imaging techniques as well as volatile organic compound (VOC) detection. Direct methods, on the other hand, analyze the properties of the pathogen itself either through serological techniques or nucleic acid-based methods (Sanati Nezhad, 2014; Fang and Ramasamy, 2015; Buja et al., 2021). Although nucleic acid-based techniques are regarded as the most sensitive and reliable way to diagnose pathogens (Barken et al., 2007; Lau and Botella, 2017; Prabhakar and Lakhanpal, 2020; Buja et al., 2021), their effectiveness is subjected to adequate DNA extraction procedures, which are generally time-intensive, often requiring specialized staff and reagents that are not easily portable, let alone suitable for spaceflight. These limitations are exacerbated when establishing a sustainable agricultural system away from Earth. In addition to these listed concerns, resources for synthesizing primers to perform polymerase chain reactions (PCRs) to detect suspected pathogens in space are nonexistent. Molecular laboratory facilities to perform other routine validation processes are limited and challenging in reduced gravity environments (Wong, 2020). Thus, there is a critical need for portable, automated plant disease diagnostic systems that do not require prior knowledge or predictions about the causative agents and can be used in remote environments (Haveman and Schuerger, 2022).

The current operations for analyzing microbes or microbiomes of plants grown in space require freezing the samples and sending them back to Earth for processing in the laboratory via a culture-base method or manually extracting microbial DNA for Illumina sequencing (Khodadad et al., 2020). In this study, we demonstrate a novel method that allows for the automation of extracting plant pathogen nucleic acids for downstream diagnosis with nanopore sequencing. Claremont BioSolutions LLC (CBIO) has developed several commercially available devices and reagents that enable compact field-portable automation of the nucleic acid extractions from various tissue types, except for plant tissues (Chargin et al., 2016; Hoover et al., 2016; Young et al., 2018; Pantazides et al., 2021). In fact, the WetLab-2 team at NASA Ames Research Center (Moffett Field, CA) has previously worked with CBIO to develop proprietary reagents and hardware for microbial RNA extractions that are spaceflight-approved (Parra et al., 2017). Using some of the CBIO technologies, we developed a method that enables the simultaneous extraction of plant and plant pathogen DNA and is well suited for use with the MinION sequencing platform for diagnosis currently available on the International Space Station (ISS). We tested this method with three different plant hosts and phytopathogens (henceforth called pathosystems) to show the versatility of the methods for future adaptation for spaceflight utilization.

## 2 Materials and methods

### 2.1 *Arabidopsis* and *Escherichia coli* tests

*Arabidopsis thaliana* (*Arabidopsis*) plants were grown on 0.5% Phytagel/0.5×MS media within Magenta Vessels (Sigma-Aldrich Chemical Co., St. Louis, MO, USA). Plants were grown under a broad-spectrum light-emitting diode (LED) light bank (100 µmol/m^2^) at 22°C +/− 2°C. After large leaf rosettes developed (approximately 30 days), single *Arabidopsis* leaves were either infiltrated with *Escherichia coli* (*E. coli*) or harvested. Those that were not infiltrated were mixed with approximately 50 µl of *E. coli* pellet. Combinations of these samples were then used for the optimization of the CBIO DNA extraction protocols with various lysis buffers. For each step of the extraction process, eluants were collected and used for PCR screening. Specific primers for universal bacterial 16S rRNA and *Arabidopsis*-specific heat shock protein 70 (HSP70) were used to determine whether the DNA came from plants or bacteria.

### 2.2 Plant growth and inoculations

Plants were grown under red, blue, green, and white LED arrays that delivered approximately 275 µmol m^−2^ s^−1^ photosynthetic active radiation (PAR; 400–700 nm) in a 12:12 diel cycle. The LED arrays were purchased from Fairchild Tropical Botanic Garden (Coral Gables, FL, USA). The red/green/blue/white LED bands were set at ratios of approximately 150:40:20:125. All LED arrays were installed in microbial incubators set at the temperatures indicated below for each plant species. The three plant hosts chosen were crops that have either been grown on the ISS or are actively studied at Kennedy Space Center so that the developed procedure can be applied to future spaceflight experiments. The corresponding pathogens were selected based on either prior experience handling or accessing the pathogen. Each pathosystem was tested separately to avoid cross-contamination of the phytopathogens among diverse crops.

### 2.3 Pathosystem #1 lettuce-*Pythium*

Lettuce seeds (*Lactuca sativa*) cv., ‘Outredgeous Red’ (Johnny’s Selected Seeds, Winslow, ME, USA), were propagated into autoclaved silica sand and irrigated with a half-strength modified Hoagland’s nutrient solution (Schuerger and Mitchell, 1992). Plants were grown under LED arrays for 21 days and inoculated with the fungal phytopathogen, *Pythium aphanidermatum* (strain P1717; from Erica Goss, Dept. of Plant Pathology, Univ. of Florida, Gainesville, FL, USA). Lettuce canopies were incubated at 24°C +/− 1°C; and *P. aphanidermatum* cultures were maintained on V-8 agar (Dhinga and Sinclair, 2000) for 48 h prior to root inoculations.

Lettuce root systems were inoculated by boring two 1-cm-wide holes in the silica sand to the depths of the Magenta Vessels (approximately 5 cm), placing six individual 0.8-cm agar discs of *P. aphanidermatum* mycelia into each bore-hole, and covering the holes with silica sand from the same Magenta vessel. Fungal mycelia were allowed to ramify through the sand interstitial spaces and infect roots. Infected lettuce roots were harvested at 21 days postinoculation by washing away the silica sand in autoclaved 600 ml glass beakers using sterile deionized water. The roots were then processed for the DNA extraction described below.

### 2.4 Pathosystem #2 tomato-*Fusarium*

Tomato seeds (*Solanum lycopersicum*) cv., ‘Red Robin’ (Totally Tomatoes, Inc., Randolph, WI, USA), were propagated on 0.5% Phytagel/0.5×MS media in Magenta Vessels. Plants were grown under a broad-spectrum LED light bank (100 µmol/m^2^) at 22°C +/− 2°C.

Tomato seedlings were allowed to develop for approximately 21 days until true leaves were observed. *Fusarium oxysporum* f. sp. *lycopersici* (FOL) Race 2 cultures were grown on potato dextrose agar (PDA) plates, and 5 mm agar discs were punched-out from the edge of the actively growing colony and placed on a wound site on the crown of the tomato seedlings. Symptoms were allowed to develop for 7 days, and inoculated plants showing wilting and root discoloration were harvested. Roots, stems, and leaf tissues were processed separately for DNA extraction, as described below.

### 2.5 Pathosystem # 3 pepper-ToMV

Pepper seeds (*Capsicum annuum*) cv., ‘Chablis’ (Totally Tomatoes Inc., Randolph, WI, USA), were propagated in autoclaved (45 min at 121°C and 1.1 kg cm^−2^) silica sand within Magenta Vessels and irrigated with a half-strength nutrient solution (Schuerger and Mitchell, 1992). Pepper plants were grown at 28°C +/− 1°C.

Pepper plants were allowed to develop until canopies were composed of between 8 and 10 fully expanded true leaves (approximately 28–32 days), and then two lower-canopy true leaves per plant were inoculated with tomato mosaic virus (ToMV) (obtained from Scott Adkins, United States Dept. of Agriculture, Ft. Pierce, FL, USA). ToMV symptoms were allowed to develop for 5 days, and inoculated leaves showing local lesions were harvested. Systemic symptoms were observed between 7 and 10 days. The upper true leaves in the canopies with systemic symptoms were harvested at 14 days postinoculation. All ToMV-infected pepper leaf tissues were processed for RNA extraction, as described below.

### 2.6 DNA/RNA extraction with Claremont BioSolutions kit

Plant materials were harvested, weighed, and diced before being placed into CBIO microHomogenizer™ 2 ml tubes. Lysis beads, prefilter columns, fast-flow nucleic acid binding columns, PureLyse® 8× CBBB binding solution, and PureLyse® 1× CBBB wash solution were obtained from Claremont BioSoluitons (Upland, CA). For DNA extractions, 1.1 g of CBIO lysis beads, lysis buffer (0.2 M Tris-HC1 [pH 8], 10 mM EDTA [pH 8], 0.5 M NaC1, 1% SDS), and 20 µl of Proteinase K (catalog # 19131, Qiagen, Hilden, Germany) were added to the CBIO microHomogenizer^™^ 2 ml tubes and homogenized for approximately 10 min at room temperature. Lysate was filtered through the CBIO prefilter column using a 3-ml syringe and incubated with 10 µl of RNase A (catalog # 19101, Qiagen, Hilden, Germany) at room temperature for 5 min. Precipitation of nucleic acids in solution was performed by adding 500 µl of isopropanol (ThermoFisher Scientific, Waltham, MA, USA) to the filtered lysate. A 1:1 volume ratio of CBIO 8× CBBB binding solution was added to the sample-isopropanol mixture and the entire volume was then loaded onto the CBIO binding column. The binding column was washed twice with 4 ml of CBIO 1× CBBB wash solution and purged with air using a 5-ml syringe. Elution buffer (10 mM Tris, 1 mM EDTA [pH 8]) of 200 µl was then added to the column, incubated for 2 min, and collected. For RNA extractions, an additional 40 µl dithiothreitol (DTT) was added to the lysis buffer and the RNase A incubation step was eliminated.

To evaluate the presence of phytopathogen nucleic acids in the inoculated samples, species-specific PCR tests were performed. Specific primers (**Table 1**) were used for each of the phytopathogens. The PCR amplification of each of the listed primers was performed as described in the respective references (**Table 1**). For HSP70 (AT1G09080), the PCR was carried out with an initial denaturing step at 95°C for 5 min, followed by 35 cycles of denaturing at 95°C for 30 s, annealing at 55°C for 30 s, and extension at 72°C for 30 s. The final extension was performed at 72°C for 10 min. From all the DNA extracted, 4 μl was used as a template for each PCR reaction. RNA extracted from the pepper–tomato mosaic virus pathosystem was first converted into cDNA using the high-capacity RNA-to-CDNA kit (Applied Biosystems, Waltham, MA, USA) according to manufacturing guidelines before PCR was performed.

**Table 1:** Sequences of forward and reverse primers used during PCR.

Target organism	Primer sequence (5′–3′)		Product size (bp)	Reference
	Forward	Reverse		
16srRNA (799F + 1193R)	AACMGGATTAGATACCCKG	ACGTCATCCCCACCTTCC	394	Thijs et al. (2017)
HSP70 (AT1G09080)	CAAGGAAAACACAGCGAAGATG	CTATCACCGTCCCCAGTTTC	210	This study
*Pythium aphanidermatum* P1771	AACCCCGACTTCAGACAATG	GCCCTCGAACCACCACCACAC	656	Zadeh et al. (2014)
*F. oxysporum* f. sp. *lycopersici* (Fol) Race 2	CCAGCCAGAAGGCCAGTTT	GGCAATTAACCACTCTGCC	608	Carmona et al. (2020)
Tomato Mosaic Virus (ToMV)	AAGATGTCAAACCAACTTTA	GAAACATCCAACTCAAGTACG	595	Sui et al. (2017)

### 2.7 Nanopore DNA-PCR library preparation

For nanopore sequencing, the eluted DNA samples were cleaned and concentrated (catalog # D4011, Zymo, Irvine, CA, USA) to ensure compliance with nanopore’s quality and quantity guidelines. The DNA-PCR library preparation was performed using the Rapid PCR Barcoding Kit (SQK-RPB004) according to the manufacturer’s instructions (Oxford Nanopore Technologies, Oxford, UK). Libraries for barcodes 1–4 and 5–6 were prepared separately and run on separate flow cells. Sequencing was performed on a MinION Mk1C device using MinION FLO-Min106 R9.4 version flow cells (Oxford Nanopore Technologies, Oxford, UK). Barcodes (BC) used in this study are reported in **Table 2**.

**Table 2:** Barcodes and samples used.

Samples	Barcode for nanopore sequencing
CBIO healthy tomato stem	BC01
CBIO FOL-inoculated tomato stem	BC02
CBIO FOL-inoculated tomato roots	BC03
CBIO healthy tomato root	BC04
CBIO healthy tomato leaf	BC05
CBIO FOL-inoculated tomato leaf	BC06
CBIO healthy lettuce roots	BC07
CBIO *Pythium*-inoculated lettuce roots	BC08
CBIO *Pythium*-pure culture	BC11

### 2.8 Bioinformatics and data analysis

Sequencing data was based-called in real-time using the MinKNOW software v21.11.6 via the MinION Mk1C device. All FASTQ files were concatenated, and sequencing statistics were analyzed using Nanoplot v1.0.0 (De Coster et al., 2018). Barcodes were demultiplexed and adapters were trimmed via the MinKNOW software v21.11.6. Reads (**Table 3**) were filtered for a minimum read length of 500 bp and a minimum *Q*-score of 10 using Filtlong v0.2.0 (Wick, 2020). Taxonomic classification of genomic DNA reads was performed with Kraken2 v2.0.8b (Wood et al., 2019) using a modified Kraken2-microbial database (https://lomanlab.github.io/mockcommunity/mc_databases.html).

**Table 3:** Summary statistics for nanopore sequencing.

	FC1	FC2	
Statistics for metatranscriptome sequences			
Mean read length (bp)	2,628.8	2,747	
Read length N50 (bp)	2,884	3,045	
Mean read quality (Q score)	10.7	10.2	
Percentage of reads > Q score 10	90.10%	88.60%	
Total bases (bp)	9,882,459,549	8,646,383,589	
Total number of reads	9,430,127	7,889,956	
Total Reads after filtlong QC^a^ (min length > 500bp)	3,098,543	2,178,538	
^a^Quality control (QC) threshold of Q score < 10 and read length of < 500 bp were excluded in the downstream analysis.			
Barcodes 5 and 6 were run on FC 1 and barcodes 1-4 were run on FC2			Total number of reads after QC
BC01 – CBIO Healthy Tomato Stem			982,250
BC02 – CBIO FOL Inoculated Tomato Stem			320,626
BC03 – CBIO FOL Inoculated Tomato Roots			175,019
BC04 – CBIO Healthy Tomato Root			700,667
BC05 – CBIO Healthy Tomato Leaf			1,851,710
BC06 – CBIO FOL Inoculated Tomato Leaf			1,246,833

## 3 Results and discussion

### 3.1 Optimizing nucleic acid extraction for downstream plant disease diagnosis

Extracting high-quality DNA from plant material is not a trivial exercise. Complex cell wall macromolecules (e.g., cutin and lignin) can be difficult to homogenize, and proper care is needed to ensure the removal of potential plant-associated inhibitors (e.g., polysaccharides and phenolic compounds), as these compounds can have repercussions on downstream assay’s performance (Wilson, 1997). The conventional protocols for plant nucleic acid extractions require equipment and reagents that are not easily portable. POC diagnostic assays on Earth and in space will require the development of simple, portable, and affordable methods of extracting DNA. However, for spaceflight applications, automation will also be an important criterion. Although several methods have been suggested as potential techniques for POC DNA extraction applications (Lau and Botella, 2017), none currently have the automation capability needed for spaceflight applications.

**Figure 1:**
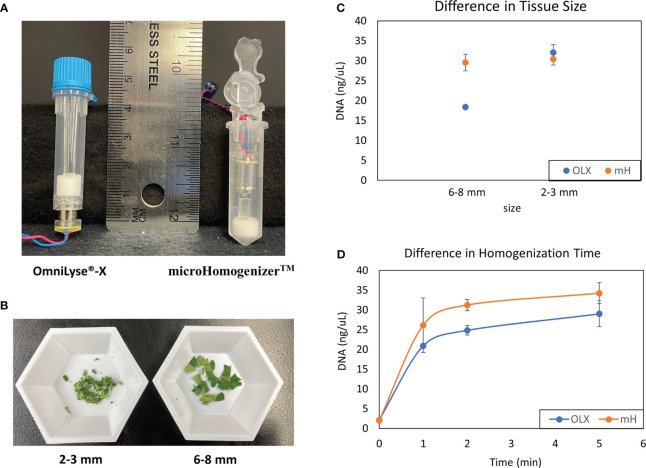
Comparison of mechanical disruption of plant tissues with CBIO devices. **(A)** Disposable, battery-operated OmniLyse® tube and microHomogenizer^™^ devices. **(B)** Sizes of diced-up tomato leaf tissue (2–3 and 6–8 mm) were used to determine cell lysis efficiency. **(C)** Comparison of leaf tissue disruption and cell lysis between both the OmniLyse® and microHomogenizer^™^ devices using different sizes of leaf tissue (6–8 and 2–3 mm). DNA concentrations were measured and recorded. Three replicates were performed for each data point. **(D)** A homogenization time course study using 0.4 g of 3 mm lettuce leaf punches and DNA concentrations was completed. Three replicates were performed for each data point. TE buffer was used so that DNA concentrations from lysate within the devices after homogenization could be measured with Qubit^™^ dsDNA broad range (BR) kit.

To isolate nucleic acids from plant samples with the ability for future automation, we optimized a protocol with potential compatibility with CBIO’s SimplePrep® automated platform, which allows integrated, cartridge-based lysis and DNA extraction from hard-to-lyse samples. For the homogenization of plant tissues, both CBIO OmniLyse®-X and microHomogenizer^™^ devices (**Figure 1A**) were tested. Tomato leaf tissues, approximately 0.2 g, were placed into the respective tubes with the TE buffer, and beads, and allowed to homogenize for 10 min. Variations in the size of leaf tissues used in the tubes were also tested (**Figure 1B**). Assessment of the level of tissue disruption from both devices was performed through quantification of accessible DNA within the lysate. Both devices were generally able to disrupt leaf tissues and break open cells. However, results from the microHomogenizer^™^ device showed the ability to break open cells slightly better than the OmniLyse®-X device (**Figure 1C**; **Supplementary Table S1**) when leaves were about 6–8 mm in size. The microHomogenizer^™^ device’s design enabled the leaf tissues to be compressed to the bottom third of the tube, allowing the beads and the rotor to have constant contact with the bigger-sized leaf tissue breaking open the cells more efficiently. In addition, using 0.4 g of 3 mm lettuce leaf punches per device (*n* = 3), a homogenization time course was performed for both the OmniLyse®-X and microHomogenizer^™^ devices. Results showed similar DNA yields after 5 min of homogenization in TE buffer (**Figure 1D**; **Supplementary Table S1**). Having more flexibility with tissue size, the microHomogenizer^™^ device was used to perform all experiments in this paper.

**Figure 2:**
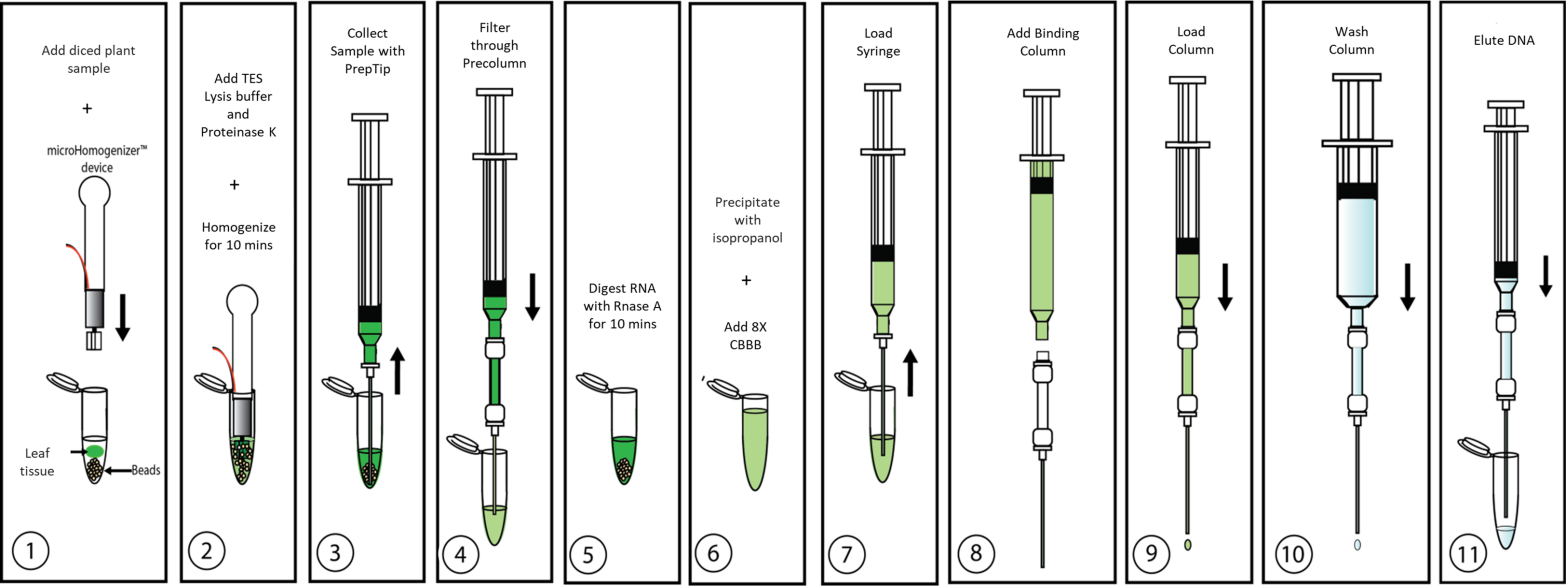
Overview of CBIO DNA extraction protocol. Details are described in “**Plant growth and inoculations**”.

**Figure 3:**
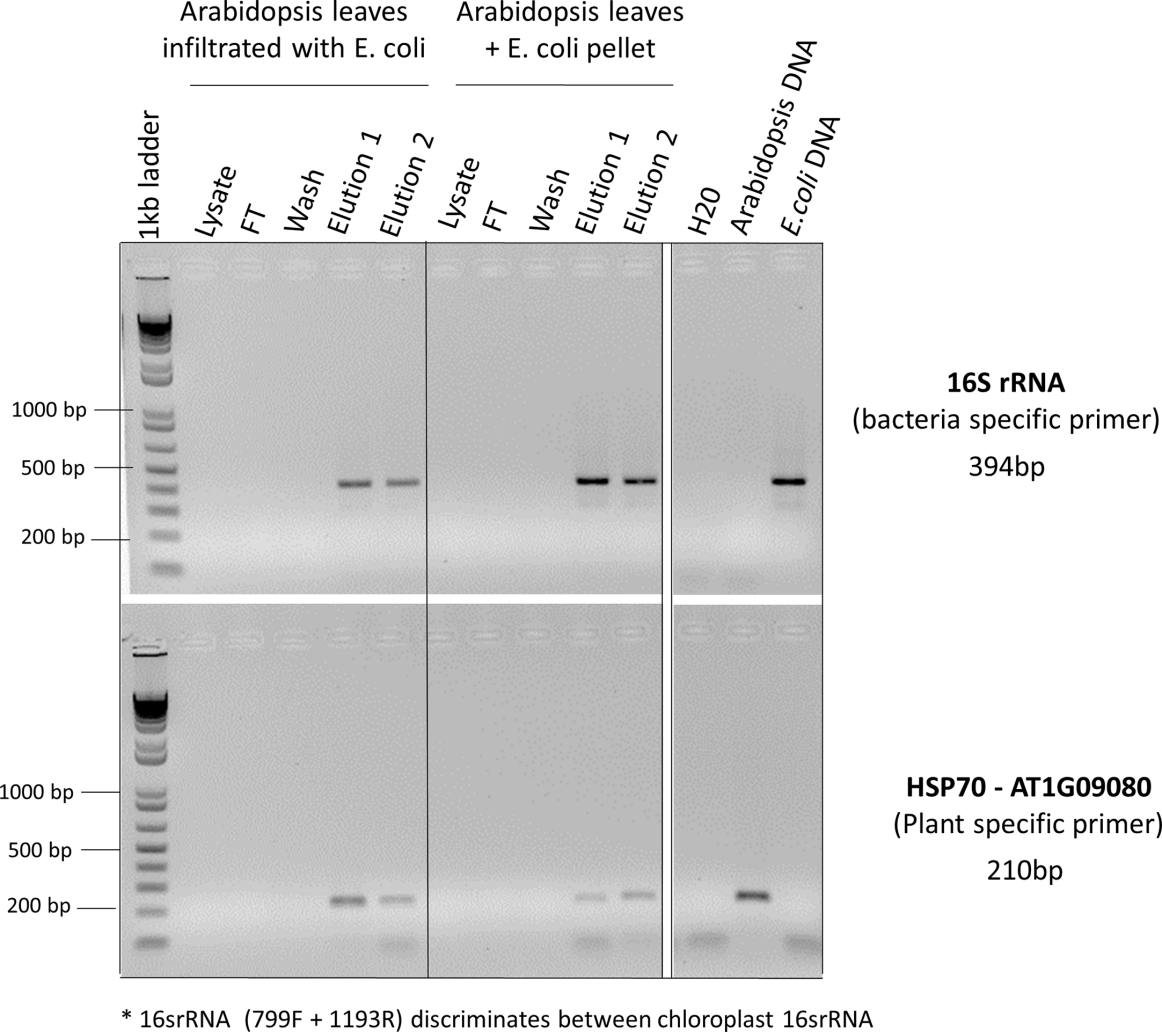
PCR reactions to determine whether both plant and microbial DNA were extracted. *Arabidopsis* leaves infiltrated with *E. coli* or just mixed with *E. coli* pellets were used for CBIO DNA extractions. For each step of the protocol (lysate, flow though (FT), wash, elution 1, and elution 2), eluants were collected and used for PCR screening. Specific primers for universal bacterial 16S rRNA and *Arabidopsis*-specific heat shock protein 70 (HSP70) were used to determine whether the DNA was coming from plants or bacteria. ^*^The 16SrRNA primers used in this PCR reaction (primers 799F + 1193R, **Table 1**) discriminate between chloroplastic 16SrRNA and bacterial 16SrRNA. *Arabidopsis* leaves mixed with *E. coli* pellet, as well as pure *Arabidopsis* DNA and pure *E. coli* DNA served as a positive control.

Identifying the best lysis buffer compatible with the SimplePrep® Technology required extensive testing of standard plant nucleic acid extraction buffers. Some lysis buffers (i.e., with CTAB and/or PVP components) resulted in the lysates becoming too effervescent to pull through the filter with a piston; others (i.e., with the addition of plant enzymes like cellulase, Macerozyme, and/or pectinase as well as Edwards buffer or an EDTA-EGTA-Tris-HCL combination) resulted in the extraction of poor-quality genomic DNA. The best lysis buffer tested was the TES buffer (0.2 M Tris-HC1 [pH 8], 10 mM EDTA [pH 8], 0.5 M NaC1, 1% SDS) that has been previously used to extract DNA from a range of pathogens and plants without the use of toxic and hazardous reagents (Mahuku, 2004). The optimization of the protocol illustrated in **Figure 2** and detailed in the methods and material section shows how this simple protocol can be readily incorporated into the SimplePrep® automated system. To determine whether both plant and microbial DNA were extracted with this protocol, specific primers for universal bacterial 16S rRNA and *Arabidopsis*-specific heat shock protein 70 (HSP70) were used in a PCR reaction (**Figure 3**). DNA extracted from *Arabidopsis* leaves infiltrated, or just mixed, with *E. coli* showed that both plant and microbial DNA are obtained with this protocol (**Figure 3**). Thus, apart from diagnosing plant diseases, this method of extraction can be used to isolate plant DNA for downstream molecular investigations in the spaceflight environment.

### 3.2 Three different pathosystems show the versatility of nucleic acid extraction methods to detect phytopathogens

To test the ability to use this nucleic acid extraction method for downstream plant disease diagnoses, we set up three different pathosystems with plant species commonly used in spaceflight experiments. Three different plant species were grown to mid-harvest stages and included lettuce, tomato, and pepper. These plants were subjected to the inoculation of the respective phytopathogens, *Pythium aphanidermatum* P1771, *Fusarium oxysporum* f. sp. *lycopersici* (FOL) Race 2, and ToMV.

The first pathosystem tested in this study was lettuce-*Pythium.* The genus *Pythium* is a group of mycelium-containing organisms classified as oomycetes. Although not all *Pythium* species are pathogenic, many members of this genus cause destructive diseases on crops worldwide (Bodah, 2017). Specifically, *P. aphanidermatum* is well known to cause root rots in hydroponically grown or nutrient-film-grown lettuce cultivars (Utkhede et al., 2000; Koohakan et al., 2008; Pinto et al., 2011; Talubnak et al., 2022). Thus, this pathosystem may be relevant to future space crop applications since it is anticipated that future greenhouses on the Moon and Mars would likely adopt controlled agricultural techniques such as hydroponics, aeroponics, or some combination of the two.

**Figure 4:**
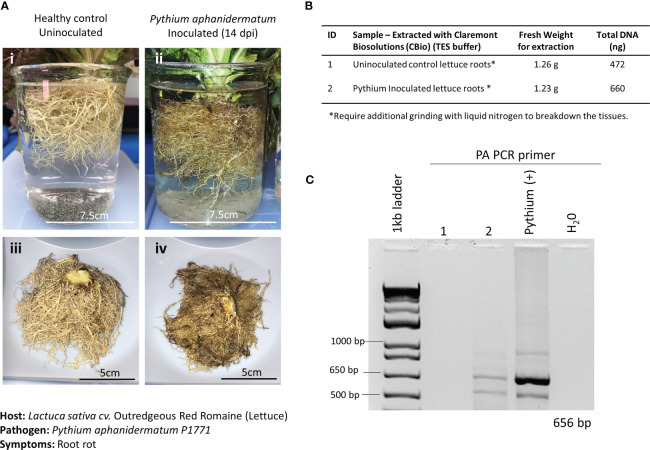
Pathosystem #1 lettuce-*Pythium*. **(A)** Images of healthy Outredgeous red romaine lettuce roots (i, iii) compared with *Pythium aphanidermatum*-inoculated roots 14 days postinoculation (dpi) (ii, iv). **(B)** Fresh weights of healthy and inoculated plant material used for DNA extractions along with the respective total DNA yields. **(C)** PCR gels showing targeted 656 bp amplified fragments for the detection of *P. aphanidermatum* using primers listed in **Table 1**.

In the current study, 21-day-old lettuce roots were inoculated with *P. aphanidermatum*, and symptoms were allowed to develop for 14 days postinfection (dpi). Although healthy lettuce control plants were grown simultaneously, they were placed in a different incubator from the infected plants to prevent potential cross-contamination. Symptoms of the infected lettuce roots were extensive and composed of dark moribund roots and significant tissue necrosis (**Figure 4A**). Both healthy roots and infected roots were harvested and weighed. For both healthy and inoculated lettuce roots, a fresh weight of approximately 1.2 g was used for the DNA extractions (**Figure 4B**). To aid with properly lysing plant cells within the thick and hard-to-lyse root tissues, root samples were first ground to powder using liquid nitrogen (LN2) before being placed in separate microHomogenizer^™^ devices. Although this step is not the preferred method of treating plant material in microgravity, it was necessary to macerate the thick root material for the lysis buffer to break open the plant cells and access the genomic content efficiently. Softer tissues, like leaves, did not require this additional step. In addition, plant digestive enzymes used to make protoplasts were also tested to break down the fibrous tissues for access to the genomic material. Unfortunately, these attempts did not yield good-quality genomic DNA (data not shown). Thus, moving forward, a redesign of the microHomogenizer^™^ device would be required to effectively homogenize thick, hard, and fibrous plant material without the need for an additional LN2 maceration step.

A pair of semi-specific primers designed to differentiate *P. aphanidermatum* from other Pythium isolates (Zadeh et al., 2014) were used to determine the presence of *P. aphanidermatum* in the extracted DNA. Only the inoculated lettuce roots and the positive control (DNA from cultured *P. aphanidermatum*) showed the 656-bp amplified fragment (**Figure 4C**), validating the ability to detect this plant pathogen with the developed method of extracting DNA.

**Figure 5:**
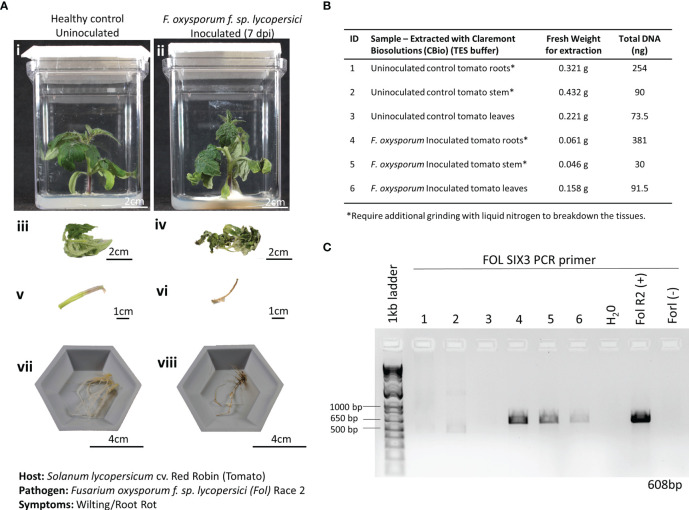
Pathosystem #2 tomato-*Fusarium*. **(A)** Images of healthy Red Robin tomato plants (i, iii, v, vii) compared with *Fusarium oxysporum f.* sp. *Lycopersici*, race 2 (FOL) inoculated roots 7 days postinoculation (dpi) (ii, iv, vi, viii). **(B)** Details of the fresh weights of plant materials used for DNA extractions and the respective total DNA yields for each sample. **(C)** PCR gels showing targeted 608 bp amplified fragments for detection of the SIX3 FOL pathogenic genes using primers listed in **Table 1**. FOL R2 (+): DNA extracted from a pure culture of FOL was used as a positive control. *Fusarium oxysporum f.* sp. *radicis-lycopersici* (FORL) is a different strain of *Fusarium* that serves as a negative control in the PCR reaction. The inclusion of FORL was to show the specificity of the *SIX3* gene to the FOL strain.

A recent plant disease outbreak identified on the ISS was caused by the opportunistic phytopathogen *Fusarium oxysporum* infecting *Zinnia hybrida* plants (Massa et al., 2017; Schuerger et al., 2021). Although the species of *F. oxysporum* isolated on the ISS was different from the one used in this study, identifying the ability to detect a fungal disease using this method of extracting DNA is critical, as many of the expected plant pathogens in space-based BLSS modules fall within the fungal kingdom. Here, 3-week-old Red Robin tomato seedlings grown in Magenta Vessels were inoculated with *F. oxysporum* f. sp. *lycopersici*, Race 2. Wilting and root discoloration symptoms developed after 7 dpi, and leaf, stem, and root tissues were harvested and weighed from both the infected and healthy plants (**Figure 5A**). The tissues of the infected plants had chlorosis, necrosis, and wilt, resulting in a substantially reduced fresh weight when compared to the tissues harvested from the healthy plant (**Figure 5B**). Of note, the root and crown tissues of the infected plants were extensively covered in FOL mycelia at the time of harvest. In the FOL pathosystem, smaller amounts of plant tissues (average of 0.2 g) were harvested to also test the limits of how much plant material was needed to obtain sufficient DNA for diagnosis. Just like the lettuce-*Pythium* pathosystem, thick, fibrous, and hard-to-lyse tissues, like roots and stems, needed to be further ground in LN2 before being placed in the microHomogenizer^™^ (**Figure 5B**). DNA extracted from the various tissue types showed only amplification of FOL in the infected tissues and not in the healthy tissues (**Figure 5C**).

**Figure 6:**
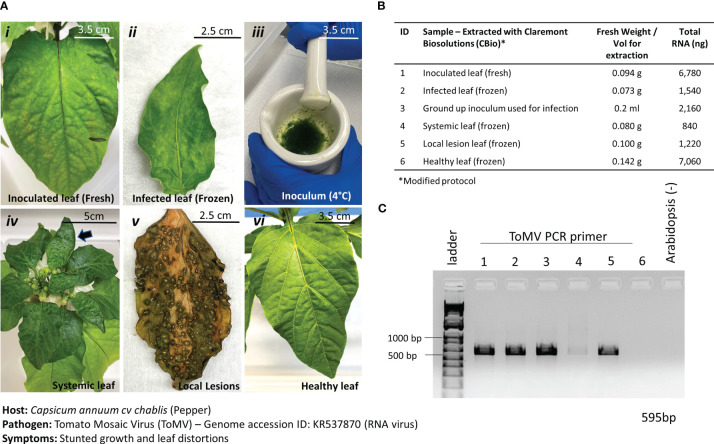
Pathosystem #3 pepper-ToMV. **(A)** Images of the various stages of Tomato Mosaic Virus (ToMV)-infected pepper leaves (i, ii, iv, v) compared to healthy pepper leaves (vi) and the original inoculum used to infect the peppers. A comparison between fresh leaf tissue **(i)** and frozen leaf tissue **(ii)** was done to identify the best method of processing the tissues. **(B)** Details of the fresh weights of plant materials used for RNA extractions and the respective total RNA yields for each sample. Modified TES buffer and protocol were used for RNA extraction. **(C)** PCR gel showing targeted 595 bp amplified fragment for detection of ToMV using primers listed in **Table 1**. Healthy *Arabidopsis* leaves were used as a negative control in the PCR reaction.

ToMV is a plant pathogenic virus found worldwide and affects tomatoes and many other plants. ToMV is an RNA virus that causes plants to be stunted and chlorotic, develop leaf distortions, and develop early necrosis of leaves. ToMV is also systemic in the host, and thus, the detection of the viral presence can also be done in young, developing leaves with symptoms of distortion. The ToMV pathosystem was incorporated into this study to test the versatility of the nucleic acid extraction protocol to extract RNA. In addition, frozen versus fresh leaf samples (**Figure 6A**, i and ii) were tested to inform best practices of harvesting samples for diagnoses. Leaves with varying symptoms of ToMV infections were also tested (**Figure 6A** i–v) for effective diagnoses. By only slightly modifying the protocol—adding a reducing agent (i.e., DTT) into the lysis buffer and removing the RNase A incubation step—we were able to extract sufficient RNA from the leaves (**Figure 6B**). The addition of the reducing agent is necessary to break disulfide bonds in RNase A and ensure high yields of RNA.

Results from the extractions of comparable leaf samples showed that fresh leaf tissues yielded substantially more RNA than frozen leaf tissues, as expected. PCR amplification with ToMV primers (**Table 1**) revealed a 595-bp fragment only in the inoculated leaf material and the inoculum but not in the healthy leaf tissue (**Figure 6C**). Thus, although fresh plant material yielded better results and is the preferred input material when given the option, it is anticipated that during operations on the ISS, samples would be harvested and kept frozen until a scheduled time to perform the diagnostic procedures is allotted. Results here show that the diagnosis of plant pathogens will not be impaired under such circumstances. Samples can also be harvested at various stages of disease for diagnosis.

### 3.3 Downstream nanopore sequencing can identify phytopathogens in inoculated samples

**Figure 7:**
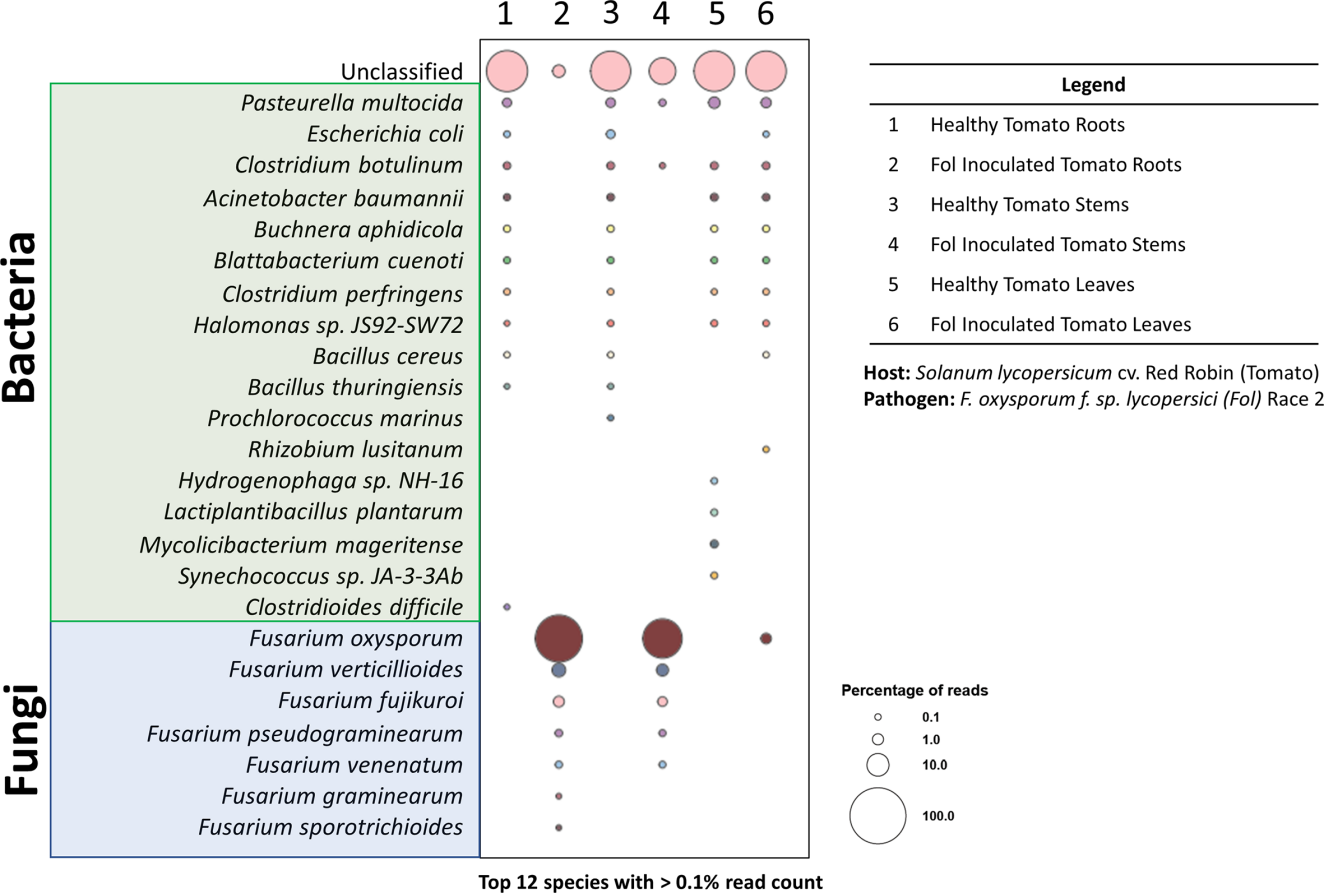
Taxonomy diversity of genomic DNA reads in plant samples from pathosystem #2 analyzed by Kraken2. Percentage of reads from the top 12 species with >0.1% reads were selected from each sample and visualized in a bubble plot. The size of the bubble indicates the percentage of reads assigned to each species. The corresponding samples for each barcode are listed in the legend. Species highlighted in green and blue belong to the bacteria and fungi kingdoms, respectively.

The unique features (i.e., portability, low cost, real-time data generation) of the MinION nanopore sequencer have enhanced the way the scientific community approaches rapid phytopathogen diagnoses (Martinelli et al., 2015; Bronzato Badial et al., 2018; Filloux et al., 2018; Chalupowicz et al., 2019; Fellers et al., 2019; Phannareth et al., 2021; Xu et al., 2021; Marcolungo et al., 2022). These same features also make the nanopore sequencer a promising candidate for future point-of-care diagnostic tools in space. DNA extracted from various tissues of the tomato-FOL pathosystem was sequenced with the MinION to see if the results obtained corroborated with those from the PCR reactions (**Figure 5C**). The tomato leaf tissues from the healthy and infected plants were run on one flow cell, while the stem and root tissues were run on a separate flow cell. The sequencing statistics showed that the average mean read lengths for both libraries were approximately 2,688 bp and mean quality scores (*Q*-scores) were > 10 (**Table 2**). Within the nanopore community, the accepted minimum *Q*-score for the R9.4.1. nanopore chemistry is 7 (Delahaye and Nicolas, 2021). Even though there were some variabilities between the samples for the numbers of reads analyzed (**Table 2**), Karken2 generated reports that summarized the percentages of reads that were assigned to specific taxons. Taxonomy classification Kraken2 reports were sorted based on the highest percentage of fragments covered by clades and the rank codes (S) for species. The top 12 species with the highest percentage of ≥ 0.1% were selected from each sample and visualized as bubble plots (**Supplementary Table S2**; **Figure 7**).

The bubble plot analysis for the tomato-FOL pathosystem (**Figure 7**) illustrated that only the FOL-inoculated samples showed the presence of the species *F. oxysporum* f. sp. *lycopersici*. Other epiphytic/endophytic microbes commonly found in/on tomatoes were seen in both the healthy and inoculated samples (Dong et al., 2019; Panebianco et al., 2022). In addition, it is likely that the unclassified reads are plant sequences. Since all sequences were mapped against the microbial database, plant sequences (**Figure 3**) were likely not classified and would be categorized as unclassified.

When harvesting the inoculated root and stem samples, it was noted that an extensive portion of the samples was covered with mycelia from the FOL agar plugs. This was reflected in the substantial proportion of reads being assigned to FOL. However, wilted tomato leaves did not have observable FOL mycelia. Thus, the smaller number of reads assigned to FOL is likely to have derived from infections through the vascular systems. Further methods, such as dissections and staining, to validate this hypothesis were not performed. Nonetheless, the results here showed that using the pipeline outlined above, phytopathogens can be rapidly diagnosed from infected tissues. This is consistent with results from preliminary tests with the MinION platform on the opportunistic phytopathogen *F. oxysporum* recovered from infected *Z. hybrida* leaves during a 2015 ISS experiment (Haveman and Schuerger, 2022). Both pathosystems #1 (lettuce-pythium) and #3 (pepper-ToMV) were not presented in the bubble plot for a couple of reasons. The intent of pathosystem #3 was to show the versatility of the extraction system—through the extraction of RNA. Thus, as the focus was on the extraction method, the appropriate RNA sequencing was not performed as it required a completely different library preparation procedure. For pathosystem #1, the challenges are due to the limitations of the database, which we will elaborate on further.

It is worth noting that not all the phytopathogens have their complete genomes available in well-curated databases (i.e., National Center for Biotechnology Information (NCBI) RefSeq), and therefore, the methodology outlined herein will initially be limited to phytopathogens with curated and accessible genomes. The standard Karken2 pipeline utilized curated genomes of organisms that were pulled from the NCBI RefSeq database. To date, of the 420 GenBank genomes within the Oomycota, the classification of only a handful of genomes (~ 8 genomes) have been curated into the RefSeq database (“NCBI Oomycota Genome, n.d.”). Although the sequenced genome *P. aphanidermatum* (Nguyen et al., 2022) has been published, it has not been curated into the RefSeq database, and there are still some disparities between proper nomenclatures within the genus. Thus, using the pipeline above, we were unable to detect *P. aphanidermatum* in the lettuce root samples as it was not found in the RefSeq database. An attempt to create a new Oomycota database from the noncurated GeneBank genomes to identify *P. aphanidermatum* was performed.

Here, we were able to show (**Supplementary Table S3**) that when aligned against just the *P. aphanidermatum* genome, only the inoculated lettuce and the pure culture had hits to that genome. However, when sequences were aligned to all 420 Oomycota genomes, *P. aphanidermatum* did not show up in the healthy samples or the inoculated lettuce samples but was aligned 100% in the pure culture. Other members of the *Pythium* genus (e.g., *P. insidiosum* and *P. plurisporium*) do show up in the inoculated lettuce roots (**Supplementary Table S4**). These results suggest there needs to be better refinement of the genomes available within the *Pythium* genus.

Ongoing initiatives to develop comprehensive phytopathogen databases have made progress (Hamilton et al., 2011; Pedro et al., 2016; Urban et al., 2020); however, the numbers of curated phytopathogen genomes within these databases are still relatively small and not easily integrated into external bioinformatics pipelines. Thus, for the intended applications described in this paper, additional efforts in developing and refining phytopathogen datasets are required to fully facilitate diagnostic efforts.

## 4 Conclusion

The work presented here demonstrates a methodology that lays the foundation for future plant disease detection in spaceflight modules, extraterrestrial habitats, and other remote exploration environments. Prompt onsite disease diagnosis is an important aspect of ensuring food safety and security for future human space missions (Schuerger, 2021). This manuscript presents a method that is compliant with several restrictions posed for spaceflight applications, including proper containment, the use of safe chemicals, and the potential for full automation. Although these restrictions are mostly enforced for spaceflight, there are many applications on Earth that would also benefit from this methodology. In addition, the versatility of the developed method showed that both DNA and RNA can be easily extracted from plant tissues, which thus allows for the potential diagnoses of a myriad of different plant diseases.

Several advances to this technology are possible. Instead of *de novo* sequencing, perhaps a panel of common pathogen amplicons (customized through nanopore’s amplicon kits) can be used with this method for onsite disease diagnostics at farms, research centers, and educational centers. Thus, it is anticipated that further improvements or alternative iterations will be made to this methodology to fit the needs of the user. One conceptual improvement that would advance this methodology for spaceflight applications would be the manufacture of larger microHomogenizer^™^ tubes (a 5-ml version is currently in development at CBIO). This will allow the accommodation of more plant material so that a substantial increase in genomic material can be obtained. Another possible improvement to this system would be altering the design of the homogenizer rotor to facilitate better disruption of fibrous tissues like roots and stems. Although further work in developing these steps for automation, as well as design improvements, is currently underway at CBIO, the method described herein lays the groundwork for advancing the automation of nucleic acid extractions of plant diseases in remote locations on Earth and in space.

## Data availability statement

The original contributions presented in the study are publicly available. This data can be found here: DOI is: 10.26030/qc04-a934 accession OSD-594.

## Author contributions

NH was responsible for the overall experimental design, sample nucleic acid extractions, sequencing, data analysis, evaluations, and writing of the manuscript. AS assisted with the overall experimental design, conducted all experiments pertaining to pathosystems #1 (lettuce-*Pythium*) and #3 (pepper-ToMV), and contributed to the final editing of the manuscript. P-LY conducted all experiments pertaining to pathosystems #2 (tomato-*Fusarium*) and contributed to the final editing of the manuscript. MB and RD assisted with the procurement of the kits from CBIO, conducted the tissue lysis optimization, and contributed to the final editing of the manuscript. A-LP and RF assisted with the data evaluations and contributed to the final editing of the manuscript. All authors contributed to the article and approved the submitted version.

## Supplementary material

Supplementary Table 1 DNA concentrations collected for comparisons between OmniLyse^®^-X tube and microHomogenizer^™^ to mechanically disrupt plant tissue.

Supplementary Table 2 Sequences from pathosystem #2 (Tomato-FOL) that were aligned with Kraken2 to RefSeq microbial (bacteria and fungi) database.

Supplementary Table 3 Sequences from pathosystem #1 (Lettuce-Pythium) were aligned with Kraken2 to only the Pythium *aphanidermatum* genome.

Supplementary Table 4 Sequences from pathosystem #1 (Lettuce-Pythium) were aligned with Kraken2 to all 420 Oomycota genomes created in a customized database.
